# Are sodium‐glucose cotransporter‐2 inhibitors safe adjunctive drugs during insulin therapy in young children with type 1 diabetes? The first case of type 1 diabetes with *SLC5A2* mutation

**DOI:** 10.1111/1753-0407.13570

**Published:** 2024-06-26

**Authors:** Mohamad Ahangar Davoodi, Mohammad Ali Daneshmand, Taraneh Rezaei

**Affiliations:** ^1^ Department of Pediatric Endocrinology Arak University of Medical Sciences, Clinical Research Development Center of Amirkabir Hospital Arak Iran; ^2^ Arak University of Medical Sciences, Teacher of Pathology in Arak Medical School Arak Iran; ^3^ Student Research Committee Arak University of Medical Sciences Arak Iran

## Abstract

Highlights

- A persistent glycosuria alongside hypoglycemia in pediatric type 1 diabetes mellitus needs further evaluation.
- Morning hypoglycemia is a limiting side effect of sodium glucose transporter 2 (SGLT2) inhibitors in children younger than 5 years old.
- *SLC5A2* mutation functioning as a SGLT2 inhibitor can result in acceptable range of glycated hemoglobin in younger children and lower required doses of insulin.

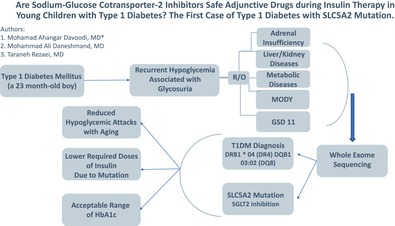

To the Editor,

Sodium glucose transporter 2 (SGLT2) inhibitors added to insulin therapy in type 2 diabetes (T2DM), have shown renoprotective and therapeutic effects on decreasing glycated hemoglobin (HbA1C), required insulin dosage, glucose intolerance, and body‐weight.^1^, ^2^, ^3^ Possible side effects of SGLT2 inhibitors include euglycemic diabetic ketoacidosis (DKA) and increased free fatty acids metabolism, making the patient susceptible to heart failure, and increased risk of urinary tract infection.^1^, ^4^, ^5^ A recently published article has shown Food and Drug Administration approval for empagliflozin (Jardiance) and empagliflozin plus metformin hydrochloride (Synjardy) for T2DM in pediatric populations aged >10 years old.^6^

SGLT2 inhibitors as adjunctive therapy in combination with closed‐loop insulin therapy can improve blood glucose control.^7^

For adults with type 1 diabetes mellitus (T1DM), the usual oral dosage is 50 mg as ipragliflozin once daily before or after breakfast, which can helpful with careful monitoring of the patient's condition.^8^

A phase 3 study to evaluate the safety of sotagliflozin in patients with T1DM who have insufficient glycemic control with insulin therapy alone has also been somewhat helpful.^9^

By reporting this case of a *SLC5A2* mutation functioning as a SGLT2 inhibitor, we hope to demonstrate the advantages and disadvantages of this drug for T1DM treatment in young children.

## CASE PRESENTATION

A 23‐month‐old boy was referred with polydipsia, polyuria, frequent postprandial hyperglycemia ranging from 220 to 467 mg.dl^−1^, normal fasting blood sugar (BS), glucosuria (3 plus), and HbA1C of 6.5%. The physical examination was normal. Without specific previous medical history, the boy had a birth weight of 4300 grams, birth height of 54 cm, and head circumference of 37 cm at birth, born to consanguineous parents with a history of hypothyroidism and hypertriglyceridemia in the mother.

He was started on insulin therapy with 0.5 unit.kg^−1^.day^−1^. Because of severe BS fluctuations accompanying fasting hypoglycemia, we reduced the insulin dose to 0.3 unit.kg^−1^ per day. Despite these measures, an episode of seizure because of morning hypoglycemia occurred. Therefore, basal insulin administration was shifted to noon and reduced to the minimum dose. Short‐acting insulin was also reduced based on insulin sensitivity factor and carbohydrate ratio with close BS monitoring. However, the patient's hypoglycemia starting after 6 h of fasting necessitated administration of raw cornstarch before nighttime sleep to prevent nocturnal hypoglycemia. An important and persistent finding was glycosuria even in the presence of patient hypoglycemia during insulin therapy. All laboratory tests included electrolytes, blood urea nitrogen, creatinine, liver function tests, ammonia, lactate, and inborn errors of metabolism panel tests were normal. Interestingly, in multiple ultrasounds, the size of both kidneys was reported to be larger than the normal age without hydronephrosis. Due to BS fluctuations, he was tested for maturity‐onset diabetes of the young (particularly type 3), and glycogen storage disease 11; both tests were negative. The evaluation of antibodies for T1DM showed a significant increase in zinc transporter 8 and a slight increase in anti‐islet‐cell antibodies, which included zinc transporter 8 Ab 413.6 U/mL (> 15 positive), anti‐insulin Ab 3.3 U/mL (>2.4 positive), glutamic acid decarboxylase Ab 4.8 U/mL (>10 positive), anti‐islet cell <1/10 (negative).

His human leukocyte antigen typing disclosed DRB1 * 04 (DR4) and DQB1 03:02 (DQ8) haplotypes which led to T1DM diagnosis. A novel homozygous *SLC5A2* mutation was found through whole exome sequencing, suggestive of familial renal glycosuria, which was subsequently confirmed through Sanger sequencing of the parents and the patient. He was also diagnosed with acquired hypothyroidism at age 6. This finding could explain frequent fluctuations of BS accompanied by persistent glycosuria in the presence of BS < 100 mg.dl^−1^.

During the 6 years of monitoring the patient, from the time of diagnosis, first weekly care, then monthly, and then quarterly, and in special conditions of the patient, such as repeated hypoglycemic attacks, even hospitalization and numerous examinations were carried out in the clinic and pediatric endocrinology and metabolism department and Amirkabir Children's Referral Hospital. Fortunately, due to the persistent and meticulous efforts of the family, all serial blood sugar charts of the patient, which were taken before and 2 hours after meals, before bed, often at 3 to 5 am with a glucometer, sometimes up to 10 times a day, were delivered to the doctor in all visits. In addition to adjusting the insulin dose based on the blood sugar chart, we practically had a complete archive of the patient's blood sugar fluctuations, which were extracted, analyzed, and reported by evaluating the statistics of the curves and paying more attention to the attacks and severity of hypoglycemia.

Fortunately, the weight and height growth chart were between the 50th and 75th percentiles (normal) and the neurocognitive‐motor development has been completely normal so far.

## COMMENTS

### Hypoglycemia

The rate of fasting and diurnal hypoglycemic attacks level 1 hypoglycemia (70 < BS < 54 mg. dl^−1^) and level 2 hypoglycemia (BS < 54 mg. dl^−1^) over 4 years of follow‐up is charted in Table 1.^10^, ^11^, ^12^

**TABLE 1:** The rate of fasting and diurnal hypoglycemic attacks level 1 hypoglycemia (70 < BS < 54 mg. dl^−1^) and level 2 hypoglycemia (BS < 54 mg. dl^−1^) over 5 years of follow‐up.

Years of follow‐up	Fasting hypoglycemia (FBS)		Diurnal hypoglycemia	
	Level 1	Level 2	Level 1	Level2
First year (2–3 years old)	15%	5%	2.51%	1.69%
Second year (3–4 years old)	9.8%	6.2%	7.1%	4.9%
Third year (4–5 years old)	7.3%	2%	3.5%	1.7%
Fourth and fifth years (5–6.5 years old)	4%	0%	2.5%	1.5%

Abbreviations: BS, blood sugar; FBS, fasting blood sugar.

**FIGURE 1:**
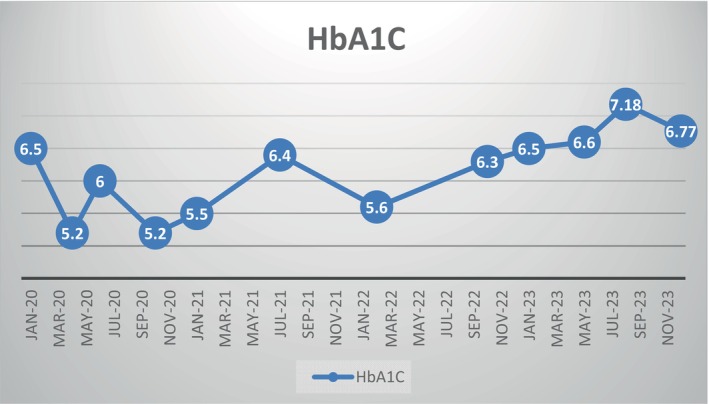
HbA1C over 4 years of follow‐up (The most notable advantage of *SLC5A2* mutation in our diabetic patient was favorable control of HbA1C). HbA1C, glycated hemoglobin.

Hypoglycemia (BS < 70 mg/dl) and serious hypoglycemia (BS < 54 mg/dl) consisted of 20% and 5% of our patient's BS chart through the first year of diagnosis (age 2–3 years old), respectively. However, they both decreased to a guideline‐approved rate (based on Sperling Pediatric Endocrinology) in the fourth year of diagnosis (Table 2).^11^ Most notably, despite the decline in hypoglycemia through the fourth year of diagnosis, HbA1C was ideally controlled (Figure 1).

Hypoglycemia is the most important risk in T1DM patients with *SLC5A2* mutation (by acting as an SGLT2 inhibitor), especially in those <5 years of age, contrary to the studies of Fattah, Evans, and Wolfs Dorf.^1^, ^4^, ^5^

### DKA

Despite the strong emphasis^4^, ^5^, ^13^ on the increased probability of ketogenesis in patients receiving SGLT2 inhibitors, leading to DKA in diabetic patients, only one episode of moderate DKA triggered by pneumonia occurred in our patient. Therefore, euglycemic DKA is not a common finding in these patients.

### Beta cell function and gluconeogenesis

SGLT2 inhibitors, possibly by reducing apoptosis and improving sensitivity to incretins secreted by beta cells, can lead to an increase in mRNA expression, MafA and Pdx1, and an increase in the active level of glucagon‐like peptide‐1, which improves blood sugar homeostasis in T1DM.^3^, ^14^ However, our patient had below‐normal C‐peptide levels during hypoglycemic attacks, which indicates that the *SLC5A2* mutation did not induce insulin secretion. Our patient's fasting hypoglycemia could be caused by a probable defect in renal gluconeogenesis, not by stimulating pancreatic beta cells to secrete insulin.

**TABLE 2:** The rate of fasting and diurnal hypoglycemic attacks (BS < 70 mg. dl^−1^) and serious hypoglycemia (BS < 54 mg. dl^−1^) over 5 years of follow‐up.

Years of follow‐up	Fasting hypoglycemia (FBS)		Diurnal hypoglycemia	
	BS < 70 mg. dl	BS < 54 mg. dl	BS < 70 mg. dl	BS < 54 mg. dl
First year (2–3 years old)	20%	5%	4.2%	1.69%
Second year (3–4 years old)	16%	6.2%	12%	4.9%
Third year (4–5 years old)	9.3%	2%	5.2%	1.7%
Fourth and fifth years (5–6.5 years old)	4%	0%	4%	1.5%

Abbreviations: BS, blood sugar; FBS, fasting blood sugar.

### HbA1C

The *SLC5A2* mutation functioning as a SGLT2 inhibitor in our patient confirms tighter HbA1C control as the most important advantage of SGLT2 inhibitor add‐on to insulin over 4 years of follow‐up (Figure 1).

### Growth

Our patient's weight and height remained in the 50th–75th percentile of the growth chart, and his neurocognitive, behavioral, and brain development remained intact despite an episode of hypoglycemic seizure.

Although optimal HbA1C and normal growth and development can be achieved, the use of SGLT2 inhibitors in children younger than 5 is limited by a higher risk of hypoglycemia, particularly after 6 h of fasting. By documenting the promising benefits of SGLT2 inhibition including tighter BS control, acceptable HbA1c range, lower required doses of insulin, and no severe hypoglycemia over the age of 5 along with normal growth and development, we hope to pave the path to greenlighting its use in children older than 5 with T1DM (Table 1, Figure 1).

## AUTHOR CONTRIBUTIONS

Mohamad Ahangar Davoodi, Mohammad Ali Daneshmand, and Taraneh Rezaei conceived and designed the study and coordinated the manuscript and the graphic abstract. Mohamad Ahangar Davoodi executed data collection, interpreted the data, and prepared the draft of the manuscript. All authors have read and approved the manuscript as it stands and are prepared to take public full responsibility for the work.

## FUNDING INFORMATION

None declared.

## CONFLICT OF INTEREST STATEMENT

The authors declare that they have no conflicting interests.

## Data Availability

All data generated or analyzed during this study will be available via contacting the corresponding author.
